# Innovative solutions to support mothers and fathers after genomic sequencing

**DOI:** 10.1038/s41431-026-02013-0

**Published:** 2026-01-13

**Authors:** Erin Turbitt, Elizabeth Callinan

**Affiliations:** University of Technology Sydney, Ultimo, NSW Australia

**Keywords:** Patient education, Ethics

Genomic sequencing is transforming paediatric care, from pilot and research studies exploring integration into prenatal and newborn screening through to routine clinical practice to diagnose neurodevelopmental conditions. This rapid expansion means more parents than ever are receiving complex genomic information about their child, often early in the illness trajectory when uncertainty and stress are high. The ability to target treatment based on genomic information can offer significant clinical benefits. The same genomic information has the potential to profoundly redefine family expectations about the future. When framed as “bad news” or deficit-focused, as frequently occurs in medical settings, these results can increase the significant emotional load already being managed by parents.

In this issue of *EJHG* Dolling et al. report on the experiences of parents following rapid genomic sequencing in neonatal or paediatric intensive care units (NICU/PICU) in England [1]. Their findings reveal persistent gaps in psychosocial support and show how these needs continue to evolve well beyond the initial diagnosis. Parents whose child received a genomic diagnosis reported poorer well-being, linked to increased medical complexity, and were more likely to feel unsupported compared to those whose child did not receive a genetic diagnosis.

While parents described receiving adequate medical care for their child, Dolling et al.’s work highlight that parents themselves often face unmet informational or emotional support needs. Many are unsure where to seek help and hesitate to burden others. Although some turn to peer or condition specific support groups, these are not universally suitable; hearing other parents’ stories can heighten anxiety about a child’s future, and more introverted parents may find such settings uncomfortable. Fathers may not recognise that support framed in emotional terms is intended for them; perceiving support presented in this way as irrelevant or not suited to their needs. Moreover, it is unreasonable and unsustainable to place the burden of psychosocial care on such support organisations, many of which are small, grass-roots initiatives that rely on philanthropic funding, highlighting the need for systemic solutions embedded within clinical pathways.

Dolling et al. state that support is widely recognised as a key protective factor in helping families adapt, representing an important target for intervention strategies. They call for a holistic and dynamic approach to meet the needs of families, including alternatives to condition-specific support groups. Innovative digital tools offer a promising solution to bridge the gaps in parental support and respond to the growing need for scalable support solutions [2]. Unlike traditional models reliant on in-person or peer group engagement, digital platforms can provide flexible options for parents who find conventional support groups overwhelming or information provided inappropriately framed.

One such example is under development in the NurtureNextGen project, though co-designing digital solutions with parents, ensuring inclusivity for both mothers and fathers and embedding flexibility to accommodate different communication styles and emotional needs [3]. Integrating such tools into genomic care pathways moves beyond ad hoc support and creates scalable, personalised systems that meet families where they are.

It is promising to see the inclusion of fathers in the work by Dolling et al., whose voices are often missing from such research. Comparing the experiences of mothers and fathers, Dolling et al offer valuable insights into how psychosocial challenges are framed. These findings will be important to inform the development of support options which are equitable and effective for all. However, the study’s sample lacked diversity in cultural and linguistic backgrounds, which limits understanding of how support needs may differ across communities. This is a critical gap, as families from multicultural backgrounds often face additional barriers to accessing information and psychosocial support [4]. Future research must include families from multicultural backgrounds and ensure that interventions are co-designed with these populations to create culturally safe, accessible and relevant support systems.

Beyond the NICU/PICU context, there is an imperative to attend to the psychosocial needs of families receiving genetic information during the perinatal period. A diagnosis during pregnancy often marks the beginning of a complex and emotional journey. Support must extend beyond clinical care to encompass emotional, informational, and practical needs. Recent initiatives such as *Facing the Unexpected* training for health professionals highlight this priority by equipping those working in this space with skills in reflective practice and interdisciplinary approaches to psychosocial care [5]. Importantly, this training has been co-designed with parents with lived experience.

As genomic medicine becomes embedded across paediatric and perinatal care, the challenge is no longer delivering results, but better understanding and attending to the psychosocial implications for parents receiving genetic information about their child and family. This means moving beyond ad hoc approaches to psychosocial care and investing in scalable, co-designed solutions that reflect the diversity of parents. Digital platforms offer one pathway, providing tailored resources and flexible engagement for parents who may not access traditional support. Initiatives that equip health professionals with the skills to better support parents are also critical. Together, these strategies can help to ensure genomic technology is used not only to provide information to families but also enabling them to receive the support and care they deserve.

## References

[1] Dolling H, Rowitch S, Bromham M, Archer S, O’Curry S, Rowitch DH, et al. Fathers’ and Mothers’ Support Needs and Support Experiences after Rapid Genome Sequencing. Eur J Hum Genet. 2025. 10.1038/s41431-025-01987-7. 41326621 10.1038/s41431-025-01987-7 PMC12858987

[2] Boulton KA, Hilton M, Sutton E, Guastella AJ. Apps and digital resources for child neurodevelopment, mental health, and well-being: review, evaluation, and reflection on current resources. J Med Internet Res. 2025;27:e58693. 39742455 10.2196/58693 PMC11736225

[3] Wu, Y, Bedi JK, Callinan E, Helwani FM, Renton H, Dray J, et al. From needs to insights: designing digital support with parents of children with genetic neurodevelopmental conditions. In: Proceedings of the 37th Australian Conference on Human-Computer Interaction. Association for Computing Machinery: Association for Computing Machinery; 2025. p. 705–19.

[4] Uebergang E, Best S, de Silva MG, Finlay K. Understanding genomic health information: How to meet the needs of the culturally and linguistically diverse community—A mixed methods study. J Community Genet. 2021;12:549–57. 34185265 10.1007/s12687-021-00537-0 PMC8554909

[5] Through the Unexpected. Facing the Unexpected. 2025; Available from: https://www.throughtheunexpected.online/.

